# Cellular immune responses induced *in vitro* by *Ehrlichia ruminantium* secreted proteins and identification of vaccine candidate peptides

**DOI:** 10.4102/ojvr.v83i1.1170

**Published:** 2016-08-30

**Authors:** Nontobeko Thema, Alri Pretorius, Selaelo I. Tshilwane, Junita Liebenberg, Helena Steyn, Mirinda van Kleef

**Affiliations:** 1 New Generation Vaccines Programme, Agricultural Research Council-Onderstepoort Veterinary Institute, South Africa; 2 Department of Veterinary Tropical Diseases, University of Pretoria, South Africa

## Abstract

Secreted proteins are reported to induce cell-mediated immunity characterised by the production of interferon-gamma (IFN)-γ. In this study three open reading frames (ORFs) (Erum8060, Erum7760, Erum5000) encoding secreted proteins were selected from the *Ehrlichia ruminantium* (Welgevonden) genome sequence using bioinformatics tools to determine whether they induce a cellular immune response *in vitro* with mononuclear cells from needle and tick infected animals. The whole recombinant protein of the three ORFs as well as four adjacent fragments of the Erum5000 protein (Erum5000A, Erum5000B, Erum5000C, Erum5000D) were successfully expressed in a bacterial expression system which was confirmed by immunoblots using anti-His antibodies and sheep sera. These recombinant proteins were assayed with immune sheep and cattle peripheral blood mononuclear cells (PBMCs), spleen and lymph node (LN) cells to determine whether they induce recall cellular immune responses *in vitro*. Significant proliferative responses and IFN-γ production were evident for all recombinant proteins, especially Erum5000A, in both ruminant species tested. Thus overlapping peptides spanning Erum5000A were synthesised and peptides that induce proliferation of memory CD4^+^ and CD8^+^ T cells and production of IFN-γ were identified. These results illustrate that a Th1 type immune response was elicited and these recombinant proteins and peptides may therefore be promising candidates for development of a heartwater vaccine.

## Introduction

Heartwater, a disease of cattle, sheep, goats and wild ruminants is caused by an intracellular rickettsiales, *Ehrlichia (Cowdria) ruminantium* (Dumler *et al*. 2001; Moshkovski 1947). Heartwater affects sub-Saharan Africa and the Caribbean where it is transmitted by ticks of the genus *Amblyomma*. The only commercial vaccine against heartwater is a live blood vaccine that requires antibiotic treatment to prevent a serious course of the disease (Van der Merwe 1987). It is, however, risky to use in non-endemic areas where the vector resides because it contains viable organisms. It also requires a cold chain for storage and distribution and does not protect against all isolates (Collins *et al*. 2003). Attempts to develop an alternative to the blood vaccine have involved inactivated (Martinez *et al*. 1994), attenuated (Zweygarth *et al*. 2005) and recombinant DNA vaccines that included genes coding for the major antigenic protein 1 (MAP1) (Nyika *et al*. 2002), low molecular weight proteins of *E. ruminantium* (Sebatjane *et al*. 2010), or a cocktail of four 1H12 *E. ruminantium* open reading frames (ORFs) (Pretorius *et al*. 2007, 2008). When a cocktail of the four 1H12 *E. ruminantium* ORFs was tested in sheep as a DNA vaccine as well as a DNA vaccine prime or recombinant protein boost, it elicited complete protection after needle challenge. However, only limited protection was achieved with these vaccines when the animals were challenged by tick infestation. Therefore, it is necessary that additional ORFs need to be identified in order to improve the efficacy of a recombinant heartwater vaccine.

Approaches used to identify vaccine candidates for recombinant vaccine development are, in general, guided by the type of immune responses that are likely to mediate protection. It is known that a cellular T helper 1 (Th1) immune response is fundamental in destruction of intracellular pathogens like *E. ruminantium* (Totté *et al*. 1999). Th1 responses are mediated by the cytokine interferon-gamma (IFN-γ), which is expressed primarily by CD4^+^ helper T cells and cytotoxic CD8^+^ T cells. Thus, identification of antigens that induce similar responses is needed for evaluation as immunogenic agents. Several *E. ruminantium* proteins have already been investigated for their ability to induce cellular immune responses *in vitro.* Five ORFs were selected from the *E. ruminantium* genome and their ability to induce proliferative responses and IFN-γ production was evaluated *in vitro* (Sebatjane *et al*. 2010). All five recombinant proteins induced proliferation of immune peripheral blood mononuclear cells (PBMCs) and IFN-γ production. The corresponding five genes were each individually incorporated into pCMViUBs, mammalian expression vector and tested as a potential vaccine in sheep using a DNA prime-protein boost immunisation regimen. A cocktail of these DNA constructs protected one out of five sheep against a virulent *E. ruminantium* (Welgevonden) needle challenge. Similarly, using a reverse vaccinology strategy, recombinant proteins that induced a cellular immune response in immune bovine and ovine PBMCs characterised by the induction of Th1 cytokines that includes, IFN-γ, iNOS, GM-CSF and TNF-α were identified (Liebenberg *et al*. 2012). These studies all used needle infected animals to screen for protective antigens. However, indications are that antigens that induce protection against natural and not artificial challenge should be identified for inclusion in a DNA vaccine. A successful DNA vaccine may therefore need to contain a combination of recombinant proteins which induce cell-mediated immunity to ensure protection against heartwater.

One of the complicating factors is that whole proteins from pathogens may contain epitopes, discrete sites recognised by lymphocytes presented in combination with major histocompatibility complex (MHC), which inhibit protective immune responses or induce immunopathology (Wang *et al*. 2005). Hence, these negative effects can be avoided if T cell epitopes that specifically stimulate immune responses are identified. Including only the protective epitopes from multiple antigens and discarding the unnecessary sequence will also allow efficient use of limited space needed to package numerous antigens in a subunit vaccine. Research directed at elucidating the epitopes of selected *E. ruminantium* proteins will provide a better understanding of which fragment of the protein is immunogenic for incorporation into a multivalent vaccine. Furthermore, to ensure that the vaccine protects under field conditions, several immunogenic epitopes that are associated with cell-mediated immunity need to be identified and characterised using mononuclear cells from tick immune animals.

In addition to several *E. ruminantium* promising vaccine targets which function extremely well as DNA vaccines under experimental conditions, secreted proteins have been shown to be of particular relevance as protective antigens against several pathogens including *Chlamydia muridarum* (Murthy *et al*. 2007), *Ehrlichia canis* and *Ehrlichia chaffeensis* (Doyle *et al*. 2006), *Mycobacterium tuberculosis* (Langermans *et al*. 2005) and *Anaplasma marginale* (Leal *et al*. 2000). These antigens have been reported to induce cell-mediated and humoral immunity characterised by the production of IFN-γ and antibodies. This indicates that secreted proteins may be potential vaccine candidates and warrants further investigation. In this study we therefore investigated whether secreted *E. ruminantium* recombinant proteins and peptides would induce similar immune responses *in vitro* by PBMC from needle infected and challenged (NI) and tick infected and challenged (TI) animals.

## Materials and methods

### Expression of recombinant proteins

*Ehrlichia ruminantium* genes of putatively secreted proteins were identified *in silico* using bioinformatics algorithms as described previously (Liebenberg *et al*. 2012). Protein expression was performed using the pET102/TOPO^®^ expression system (Invitrogen) according to the instructions of the manufacturer. Briefly, the whole *E. ruminantium* ORFs Erum8060 (0.6 kb), Erum7760 (0.75 kb) and Erum5000 (1.47 kb) as well as the equally divided four 348 bp adjacent fragments (Erum5000A, Erum5000B, Erum5000C, Erum5000D) were PCR amplified using specifically designed primers (Table 1-A1). Plasmids of the expected size were sequenced to confirm the presence of inserts and that the ORFs were in-frame. Recombinant (His_6_-tagged) proteins were purified from soluble supernatant or the inclusion bodies using the Protino^®^ Ni 150 prepacked columns kit (Macherey-Nagel) according to the instructions of the manufacturer. The purified proteins were assayed using SDS-PAGE analysis, Western blot analysis using anti-His_6_ antibodies (Roche) and heartwater immune sheep sera (Figure 1-A1, Supporting information). The recombinant proteins were acetone precipitated for use in immune assays as previously described (Van Kleef *et al*. 2002). The concentration of the recombinant proteins was determined using the Pierce™ BCA Protein Assay Kit (Thermo Scientific).

### Synthesis of peptides

Sixteen mer peptides overlapping by eight amino acids spanning the N-terminal fragment of Erum5000 (Erum5000A) (~348bp) (Table 2-A1) were synthesised by Genscript (USA) and the purity of the peptides were > 98% as analysed by high-performance liquid chromatography. The peptides were dissolved in water or 100% dimethyl sulfoxide to 1 mg/mL and stored at -20 °C. Peptides were further diluted to 100 µg/mL in complete medium prior to use in immunological assays which include lymphocyte proliferation, IFN-γ Enzyme-Linked ImmunoSpot (ELISPOT) assay and flow cytometry.

### Immunological assays

#### Inoculation of animals as source of immune mononuclear cells

Six- to eight-month-old merino sheep (s147, s6010) and three heartwater naïve Nguni cattle (b8460, b8347 and b8404) were immunised by the needle infection and treatment method and needle challenged as described previously (Liebenberg *et al*. 2012). In addition to this, to mimic field or natural immunisation, four sheep (s6355, s6821, s6822, s6823) were tick infected and treated with ticks infected with the Welgevonden strain in the laboratory as described previously (Mahan *et al*. 1998) with some modifications. Briefly, uninfected *Amblyomma hebraeum* nymph ticks were infected by feeding on a sheep that had been infected intravenously with *E. ruminantium* Welgevonden stock. Engorged nymphs were allowed to moult to adults in the laboratory. A sheep was then infected by feeding 10 adults (5 males and 5 females) heartwater infected ticks on it. The sheep was monitored daily for the onset of clinical signs and treated on the third day of febrile reaction with Terramycin^®^100 (Pfizer). The sheep were tick challenged with the Welgevonden infected ticks. Heartwater infection of ticks and sheep were confirmed by pCS20 real-time PCR (Steyn *et al*. 2008). All animal research was performed in accordance with the stipulations of the animal ethics committee at the ARC Onderstepoort Veterinary Institute and the University of Pretoria animal use and care committee and Section 20 approval from Department of Agriculture, Forestry and Fisheries.

#### Purification of peripheral blood mononuclear cells

PBMCs were purified from whole blood under sterile conditions. Briefly, blood was collected in BD (Becton, Dickinson) Vacutainer^®^- Ethylenediaminetetraacetic acid (EDTA) tubes (Becton, Dickinson) and PBMCs were isolated by density gradient centrifugation (Histopaque^®^-1077; Sigma–Aldrich^®^) as described by Liebenberg *et al*. (2012). The cells were washed three times and counted using TC10™ Automated cell counter (BioRad) and the cells resuspended (4 × 10^6^ cells/mL) in complete medium, RPMI-1640 (GIBCO^®^ RPMI + GlutaMAXTM-I) (Invitrogen) supplemented with 10% foetal bovine serum (FBS), 55 mM 2-mercaptoethanol and 1% GIBCO^®^ Pen Strep (Invitrogen).

#### Lymphocyte proliferation assay

Lymphocyte proliferation assays (LPA) were carried out in triplicate wells as described by van Kleef *et al*. (2000). Responder cells at a final concentration of 2 x 10^6^ PBMC/mL were added to respective test wells together with one of the following: *E. ruminantium* crude antigen (1 µg/mL, positive control), ConA (positive control), *E. ruminantium* recombinant proteins (10 µg/mL), synthetic peptides (10 µg/mL), Erum5000A (positive control for peptides), *E. ruminantium* recombinant protein that tested negative previously (*r*Erum4930, rNegative, negative control) or medium only (unstimulated PBMCs). Proliferation was determined by measuring the incorporation of 0.5 µCi of [methyl-3H] thymidine added during the final 18 h of the assay using a scintillation counter. Results are presented as a stimulation index (SI) ± standard deviation (s.d.), where SI is the mean counts per min (cpm) of cells stimulated with antigen divided by mean cpm of unstimulated cells. Only SI two times higher than the negative recombinant protein and *p* ≤ 0.05 was considered to be significant antigen-specific proliferation.

#### IFN-γ ELISPOT assay

The ELISPOT assay was performed as described by (Sebatjane *et al*. 2010). Briefly, PBMCs (2 x 10^6^ PBMC/mL) were seeded in ELISPOT 96 well plates (Millipore MAIPS 4510) precoated with mouse anti-bovine IFN-γ mAb CC302 coating antibody (1 μg/mL). PBMCs were stimulated with recombinant protein, peptides and controls as described for LPA. The plates were developed after 48 h incubation at 37 ºC in a humidified 5% CO_2_ incubator. The number of spots per million cells (spmc) of the antigen was compared to the number of spmc of the corresponding negative control. Only the number of spmc that were two times higher than the negative control and with a significant *p* value (*p* ≤ 0.05) as determined by Student’s *t*-test were regarded as positive. Additionally, for peptide responses only samples with at least 10 spmc were considered positive.

#### Cell surface staining

Immune PBMCs (2 x 10^6^ cells/mL) were stimulated with antigens for 48 h at 37 °C. The cells were stained with the following commercial monoclonal antibodies: CD4 (IgM, cell line GC50A), CD8 (IgG1, cell line CACT80C) and CD45RO (IgG3, cell line ILA116A) (Washington State University Monoclonal Antibody Centre, Pullman, WA) at a 1:100 dilution in PN buffer (PBS, 0.5% FBS containing 0.2% sodium azide). Following washing secondary antibodies goat anti-mouse IgM-APC (Invitrogen), goat anti-mouse IgG1-PE (Serotec) and goat anti-mouse IgG3-FITC (Serotec) were added at dilutions of 1:10, 1:40 and 1:10 respectively. All incubations were for 15 min at room temperature and washing was done twice with PN buffer. Cells were fixed with 0.2% formaldehyde in PBS. Samples were assayed on a FC 500 Beckman Coulter flow cytometer and data analysed using the Kaluza software version 1.2 (Beckman Coulter).

#### Intracellular IFN-γ staining

Immune PBMCs were purified from s6821 and s6823 for intracellular IFN-γ staining using the BD Cytofix/Cytoperm™ Kit (BD Biosciences) and protocol. Briefly cells (2 x 10^6^ cells/mL) were incubated for 72 h at 37 °C in the presence or absence of 10 µg/mL peptide or recombinant protein. Golgi stop solution was added 4 h prior to harvesting. Cells were first surface stained as described above and subsequently, intracellular IFN-γ staining was performed with fluorochrome-conjugated anti-cytokine antibody (Alexa fluor^®^488, Serotec) at a dilution of 1:20. The cells were incubated at 4 °C for 30 min in the dark, followed by washing with the supplied buffers. The cells were analysed with an FC 500 Beckman Coulter flow cytometer and data analysed using the Kaluza software version 1.2 (Beckman Coulter).

### Major histocompatibility complex typing of experimental animals

Samples of genomic DNA of animals used in this study were obtained from whole blood collected in BD Vacutainer^®^ K2E tubes containing EDTA. Genomic DNA purification was done using the Generation^®^Capture Column Kit (Gentra systems) according to the instructions of the manufacturer. Typing for Ovine MHC *Ovar*-DRB1 and BoLA-DRB3 was performed using polymerase chain reaction-restriction fragment length polymorphism (PCR-RFLP) as described by Konnai *et al*. (2003) and Van Eijk, Steward-Haynes and Lewin (1992), respectively. Briefly, the second exon of *Ovar*-DBR1 and BoLA-DRB3 was amplified by nested PCR using different primers. The resulting DNA (nested PCR product) was digested overnight at 37 °C with 5 U of either *Rsa*I, *Hae*III, *Psu*I*, Sac*I, *Sac*II, *Dde*I, *Nci*I, *Hin1*I or *Eco*RI restriction enzymes (Roche), or at 60 °C with 5 U of *Bst*NI. However, DRB RFLP patterns could not be distinguished and thus the nested PCR products were cloned to pGEM^®^-T Easy vector (Promega) and sequenced. The restriction patterns obtained were compared with published restriction maps (Konnai *et al*. 2003; Van Eijk *et al*. 1992).

### Statistical analysis

The significance of differences between immunological assay results was determined by means of the Student’s *t*-test. Differences with *p* ≤ 0.05 were considered significant.

## Results

### Expression and purification of recombinant proteins

Three ORFs (rErum5000, rErum7760, rErum8060) were chosen randomly out of a total of 24 exported proteins previously predicted by bioinformatics (Table 1, Liebenberg *et al*. 2012). They were successfully expressed in an *Escherichia coli* BL21Star™ (DE3) host strain in conjunction with the pET102/D-TOPO^®^ vector. Recombinant *r*Erum8060 and Erum7760 were purified from both the soluble (s) and insoluble (i) fractions while *r*Erum5000 was found to be insoluble and showed distinct bands at ~36 kDa, ~42 kDa and ~70 kDa respectively in SDS-PAGE and in Western blot assays that corresponded to their predicted molecular weights. In addition, heartwater immune sheep sera could detect *r*Erum7760 and -8060 in both the soluble and insoluble fractions while *r*Erum5000 could only be recognised in the insoluble fraction (Figure 1-A1, Supporting information).

**TABLE 1:** Summary of results obtained from the selection of vaccine candidates using bioinformatics tools.

Name	Closest Homologies	Base pair size (bp)	Length (aa)	pI	MW (kDa)	Signal peptides	Solubility %	Function
Erum5000	Unknown	1470	490	8.36	53.57	1–27	8.5	Exported protein
Erum7760	Lipoprotein	750	250	6.15	29.34	1–26	50.9	Exported lipoprotein
Erum8060	Lipoprotein	600	204	5.92	23.50	1–26	29.1	Exported lipoprotein

### Proliferation induced by recombinant proteins

No proliferation was induced in PBMC from the naïve s5408. Each recombinant protein induced significant proliferation in PBMC from a different sheep (Erum8060, TI s6355; Erum7760, NI s6010; Erum5000, NI s147; Table 2). Varying but significant proliferation was induced by all rproteins with PBMC from NI bovine (Table 3). When spleen cells from NI and TI sheep and NI bovine were used, Erum5000 performed the best out of the three rproteins with a SI of 21 with lymph node cells from TI s6355. All three rproteins induced the highest significant proliferation with spleen and lymph node cells from TI s6355 compared to NI s6010. Similarly, ovine spleen and lymph node cells responded with higher SI than PBMC from the same animal.

**TABLE 2:** Proliferative responses of peripheral blood mononuclear cells, spleen cells and lymph node cells from heartwater immune sheep stimulated with recombinant proteins. Immune cells were obtained from a naïve sheep (s5408), needle infected and challenged sheep (s147, s6010) and tick infected and challenged sheep (s6355).

Protein	Stimulation index for sheep number^a^							

	s5408 (naïve) (PBMC)	s147^b^ (PBMC)	s6010^c^ (PBMC)	s6010^c^ (Spleen cells)	s6010^c^ (LN cells)	s6355^c^ (PBMC)	s6355^c^ (Spleen cells)	s6355^c^ (LN cells)
rErum8060s	1.4 ± 0.1	2.4 ± 0.0	0.0	**2.9** ± 0.7	**2.1** ± 1.0	**3.0** ± 1.7	**9.4** ± 1.0	**14.9** ± 1.0
rErum7760s	1.1 ± 0.1	0.9 ± 0.4	**2.0** ± 0.6	2.5 ± 0.7	0.2 ± 0.8	0.0	**6.8** ± 0.8	**13.2** ± 1.7
rErum5000i	0.6 ± 0.1	**6.0** ± 0.1	0.5 ± 0.2	**3.0** ± 0.5	**2.3** ± 0.2	1.4 ± 0.3	**7.7** ± 0.2	**21.3** ± 0.5
rErum5000A	n/d^d^	n/d	0.5 ± 0.1	**7.0** ± 1.2	**18.0** ± 2.1	**2.2** ± 0.9	**15.0** ± 1.7	**30.4** ± 7.5
rErum5000B	n/d	n/d	0.4 ± 0.2	**5.0** ± 1.5	**12.0** ± 3.2	**2.1** ± 0.4	**12.0** ± 0.5	**18.5** ± 3.6
rErum5000C	n/d	n/d	n/d	n/d	n/d	n/d	n/d	n/d
rErum5000D	n/d	n/d	n/d	n/d	n/d	n/d	n/d	n/d
Negative rprotein	0.7 ± 0.1	1.8 ± 0.1	0.0	1.4 ± 0.2	0.8 ± 0.1	0.0	0.7 ± 0.2	4.4 ± 1.0
Positive Antigen	**2.1** ± 0.1	**4.6** ± 0.1	1.3 ± 0.4	**3.4** ± 0.4	**6.0** ± 1.8	1.8 ± 0.5	**2.6** ± 0.3	4.4 ± 1.1

a Only stimulation index values that were two times higher than the stimulation index of the negative rprotein and had significant *p* values (*p* ≤ 0.05 as determined by Student’s *t*-test) were regarded as positive and these are indicated in bold;

b Each protein was tested at a concentration of 1 µg/mL;

c Each protein was tested at a concentration of 10 µg/mL;

d n/d, not done.

PBMC, peripheral blood mononuclear cell; LN, lymph node.

### IFN-γ responses induced by recombinant proteins

No IFN-γ was induced in PBMC from the naïve s5408 (Table 4). The highest significant response was induced by Erum5000 with PBMC from TI s6822 (200 spmc) and lymph node cells from TI s6355 (272 spmc) (Table 4). This was followed by Erum8060 with PBMC from NI s147 (101 spmc) and spleen cells from TI s6355 (88 spmc). Ovine spleen and lymph node cells responded with higher spmc than PBMC from the same animal. Varying but significant proliferation was induced by Erum5000 rprotein with PBMC from NI bovine (Table 4). Erum8060 and Erum7760 only induced IFN-γ by PBMC from two bovine.

**TABLE 3:** Proliferative responses and IFN-γ production of peripheral blood mononuclear cells from infected & treated immune cattle (B8347, B8404 and B8460) stimulated with rproteins. Recombinant proteins were tested at a concentration of 10 μg/mL.

Protein	8347		8404		8460	
		
	LPA(SI ave^a^)	ELISpot(Spots/million^b^)	LPA(SI ave)	ELISpot (Spots/million)	LPA (SI ave)	ELISpot (Spots/million)
rErum5000i	**130.0** ± 2.0	27.0 ± 2.0	**17.7** ± 0.0	33.0 ± 3.0	**6.7** ± 0.4	42.0 ± 3.0
rErum5000A	**1.6** ± 0.7	32.0 ± 1.5	**12.3** ± 2.2	40.0 ± 3.0	**18.1** ± 6.4	83.0 ± 7.0
rErum5000B	**3.3** ± 1.9	22.0 ± 1.5	**17.3** ± 0.8	10.0 ± 1.0	**9.9** ± 1.2	78.0 ± 1.5
rErum5000C	**1.8** ± 0.7	13.0 ± 2.5	**7.1** ± 0.8	12.0 ± 2.1	**8.2** ± 5.2	52.0 ± 4.0
rErum5000D	**1.7** ± 0.5	13.0 ± 1.2	**7.8** ± 0.7	15.0 ± 2.7	**10.9** ± 3.3	52.0 ± 3.5
rErum7760s	**25.6** ± 0.1	52.0 ± 2.0	**4.3** ± 0.2	18.0 ± 4.0	**2.9** ± 0.8	0.0
rErum8060s	**32.9** ± 2.0	30.0 ± 1.0	**2.1** ± 0.1	33.0 ± 6.0	**2.4** ± 0.5	0.0
Negative rprotein	0.1 ± 0.2	5.0 ± 2.0	0.3 ± 0.1	5.0 ± 2.0	0.5 ± 0.3	12.0 ± 4.0
Positive Antigen	**13.0** ± 3.2	480.0 ± 5.3	**223.0** ± 10.8	675.0 ± 18.5	**346.0** ± 40.8	1523.0 ± 18.5

a The stimulation index values of rErum proteins were compared to the negative value of negative rprotein. Only stimulation index values that were two times higher than the stimulation index of the negative rprotein and had significant p values (*p* ≤ 0.05 as determined by Student’s *t*-test) were regarded as positive and these are indicated in bold;

b The number of spots per million cells of the rErum proteins were compared with the number of spots per million cells of the negative rprotein. Only the number of spots per million cells that were two times higher than the number of spots per million cells of the negative rprotein and had significant *p* values (*p* ≤ 0.05 as determined by Student’s *t*-test) were regarded positive as underlined.

LPA, lymphocyte proliferation assays; SI, stimulation index.

**TABLE 4:** IFN-γ by peripheral blood mononuclear cells, spleen cells and lymph node cells from heartwater immune sheep stimulated with recombinant proteins. Immune cells were obtained from a naïve sheep (s5408), needle infected sheep (s147, s6010) and tick infected sheep (s6821, s6822, s6823, s6355).

Protein	Number of spots/Million cells ± standard deviation for sheep number^a^										

	s5408 (naïve) PBMC	s147^b^ PBMC	s6821^c^ PBMC	s6822^c^ PBMC	s6823^c^ PBMC	s6010^c^ PBMC	s6010^c^ Spleen cells	s6010^b^ LN cells	s6355^c^ PBMC	s6355^c^ Spleen cells	s6355^b^ LN cells
rErum8060s	0.0	**101.0** ± 9.0	**38.0** ± 6.0	**92.0** ± 9.9	**60.0** ± 2.0	0.0	**67.0** ± 5.0	5.0 ± 0.0	3.0 ± 1.7	**88.0** ± 10.0	**12.0** ± 1.0
rErum7760s	0.0	0.0	**42.0** ± 1.5	**92.0** ±14.3	**57.0** ± 2.5	2.0 ± 0.6	n/d^d^	2.0 ± 0.6	0.0	**63.0** ± 6.0	**120.0** ± 20.0
rErum5000i	0.0	**81.0** ± 16.0	**43.0** ± 3.1	**200.0** ± 4.2	**58.0** ± 2.6	0.0	**42.0** ± 4.0	0.0	**5.0** ± 1.2	**69.0** ± 13.0	**272.0** ± 20.0
rErum5000A	0.0	n/d	**228.0** ± 2.1	**173.0** ±10.6	**85.0** ± 7.1	0.0	**27.0** ± 1.5	9.0 ± 0.6	**2.0** ± 1.2	**387.0** ± 6.8	**103.0** ± 2.0
rErum5000B	0.0	n/d	**150.0** ± 9.9	**15.0** ± 1.4	**30.0** ± 0.0	0.0	**13.0** ± 1.2	2.0 ± 0.6	**3.0** ± 1.7	**305.0** ± 8.6	**70.0** ± 3.5
rErum5000C	0.0	n/d	**185.0** ± 7.1	**70.0** ± 11.3	**38.0** ± 2.1	n/d	n/d	n/d	n/d	n/d	n/d
rErum5000D	0.0	n/d	**208.0** ± 0.7	**160.0** ± 17.0	**105.0** ± 17	n/d	n/d	n/d	n/d	n/d	n/d
Negative rprotein	0.0	0.0	0.0	0.0	0.0	0.0	1.4 ± 0.2	0.0	0.0	0.0	0.0
Positive Antigen	2.2 ± 0.5	2.8 ± 0.5	**320.0** ± 17.0	**480.0** ± 17.0	**158.0** ± 37.5	**10.0** ± 1.0	**60.0** ± 7.0	2.0 ± 0.6	0.0	**52.0** ± 2.9	2.0 ± 1.7

a The number of spots per million cells of the rErum proteins were compared to the number of spots per million cells of the negative rprotein. Only the number of spots per million cells that were two times higher than the number of spots per million cells of the negative rprotein and had significant *p* values (*p* ≤ 0.05 as determined by Student’s *t*-test) were regarded as positive and these are indicated in bold;

b Each protein was tested at a concentration of 1 µg/mL;

c Each protein was tested at a concentration of 10 µg/mL;

d n/d, not done.

PBMC, peripheral blood mononuclear cell; LN, lymph node.

### Expression and purification of four recombinant protein fragments of Erum5000

Because *r*Erum5000 induced significant and reproducible results, it was divided into four to provide a better understanding of which fragment of the protein is immunogenic for epitope mapping. *Ehrlichia ruminantium* ORF Erum5000 (~1.39 kb) was PCR amplified into four consecutive ~348 bp fragments (Erum5000A, Erum5000B, Erum5000C, Erum5000D) using specifically design primers (Table 1-A1). The corresponding recombinant proteins were produced in an *E. coli* Topo tools expression system resulting in Thioredoxin or His-tag fusion proteins with distinct bands at 34 kDa (Figure 1-A1).

### Proliferation and IFN-γ secretion of peripheral blood mononuclear cells s in response to four fragments of rErum5000

PBMCs from NI bovine (b8374, b8404, b8460), sheep (s147, s6010), TI sheep (s6355, s6821, s6822, s6823) and a naïve sheep (s5408) were stimulated with the four fragment proteins, Erum5000A, Erum5000B, Erum5000C and Erum5000D. All four rproteins induced immune bovine PBMCs to proliferate with SI ranging from ~2 to 18 (Table 3). In correlation with the proliferation results, PBMCs from the naïve sheep (s5408) and one NI sheep (s6010) did not produce IFN-γ after they had been incubated with proteins (Table 4). All proteins tested with immune bovine PBMCs induced significant production of IFN-γ (Table 3). The highest magnitude of IFN-γ responses was obtained when tested with TI sheep PBMCs (s6821, s6822 and s6823). Erum5000A induced the highest IFN-γ responses in all animals tested. Erum5000D and Erum5000C induced highest IFN-γ production in sheep as compared to Erum5000B. However, Erum5000B induced reproducible immune responses in both cattle and sheep. Hence Erum5000A and Erum5000B fragments were further tested for their ability to induce immune responses in spleen and lymph nodes of TI and NI sheep. Both fragments specifically induced immune cells from spleen and lymph node to secrete IFN-γ. Once again ovine spleen and lymph node cells responded with higher spmc than PBMC from the same animal.

### Peptide-specific lymphocyte proliferation and IFN-γ production

Significant proliferation and IFN-γ secretion after Erum5000A stimulation provided the rationale for mapping Erum5000A peptides for vaccine development. Thus fourteen 16-mer peptides (P1–P14) overlapping by eight amino acids were synthesised to determine the exact minimal epitope sequence that can induce recall cellular immune responses. All peptides induced significant IFN-γ with spmc ranging from ~20 to 140 when tested with PBMC from TI sheep (Figure 1) and ranged from 2 spmc to 160 spmc for cattle (Table 5). The peptides that induced the best recall responses were P9 and P14 in TI sheep and P7 and P10 in cattle.

**FIGURE 1:**
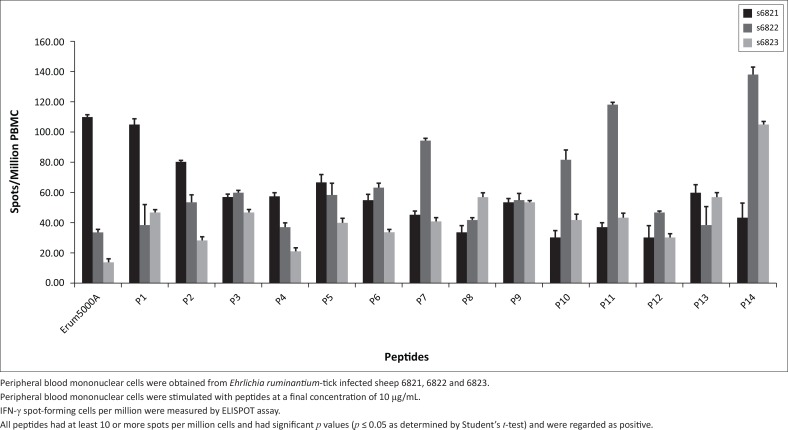
IFN-γ production by peripheral blood mononuclear cells stimulated with 16 mer overlapping peptides of Erum5000A.

**TABLE 5:** Proliferative responses and IFN-γ production of peripheral blood mononuclear cells from immune bovine (b8347; b8404 and b8406) stimulated with rprotein and peptides. Cells were stimulated with peptides at a concentration of 10 mg/mL.

Antigen	b8347		b8404		b8460	
		
	LPA SI^a^ ± s.d.	ELISpot Spmc^b^ ± s.d.	LPA SI ± s.d.	ELISpot Spmc ± s.d.	LPA SI ± s.d.	ELISpot Spmc ± s.d.
Erum5000A	**3.4** ± 0.4	15.0 ± 2.5	**3.5** ± 0.8	6.0 ± 1.8	**3.5** ± 0.8	12.0 ± 1.7
P1	**2.1** ± 0.9	22.0 ± 3.1	**2.4** ± 0.2	7.0 ± 1.2	**7.4** ± 1.0	2.0 ± 0.6
P2	1.2 ± 0.4	13.0 ± 3.1	**3.7** ± 0.2	5.0 ± 1.5	**6.4** ± 0.9	37.0 ± 7.5
P3	**2.2** ± 1.0	23.0 ± 1.5	**4.6** ± 0.4	0.0	**4.8** ± 0.5	160.0 ± 16.5
P4	1.9 ± 0.9	15.0 ± 0.0	**3.5** ± 0.4	3.0 ± 1.7	**6.0** ± 0.7	55.0 ± 5.1
P5	**2.5** ± 0.2	18.0 ± 2.1	**2.7** ± 0.4	0.0	**5.9** ± 0.6	58.0 ± 0.6
P6	1.6 ± 0.3	12.0 ± 3.2	**3.4** ± 0.2	8.0 ± 1.0	**3.4** ± 1.0	23.0 ± 2.9
P7	1.2 ± 0.3	13.0 ± 2.1	**2.0 ±** 0.04	3.0 ± 2.0	**5.0** ± 0.8	13.0 ± 3.6
P8	0.8 ± 0.7	10.0 ± 2.0	**2.4** ± 0.1	10.0 ± 1.5	**6.8** ± 0.1	43.0 ± 6.7
P9	1.7 ± 0.9	10.0 ± 2.2	**3.8** ± 0.3	3.0 ± 2.6	**7.4** ± 0.3	10.0 ± 1.7
P10	**2.7** ± 0.3	22.0 ± 2.1	**3.3** ± 0.1	2.0 ± 2.1	**5.5** ± 0.7	47.0 ± 2.1
P11	0.8 ± 0.3	12.0 ± 1.5	**4.4** ± 0.3	5.0 ± 1.5	**5.0** ± 0.8	88.0 ± 8.5
P12	0.5 ± 0.4	10.0 ± 1.7	**5.6** ± 0.6	0.0	**4.8** ± 0.7	93.0 ± 5.0
P13	1.0 ± 0.3	28.0 ± 2.5	**3.9** ± 0.2	0.0	**4.8** ± 0.3	98.0 ± 7.8
P14	1.3 ± 0.4	27.0 ± 3.1	**2.7** ± 0.4	10.0 ± 0.6	**3.8** ± 0.1	83.0 ± 7.1

a Significant stimulation index is indicated in bold and a significant increase in spots per million cells is underlined;

b Only samples with at least 10 spots per million cells and had significant *p* values (*p* ≤ 0.05 as determined by Student’s *t*-test) were regarded as positive. The stimulation index values ≥ 2 and had significant *p* values (*p* ≤ 0.05 as determined by Student’s *t*-test) were considered to be an indication of peptide-specific proliferation.

LPA, lymphocyte proliferation assays; SI, stimulation index; spmc, spots per million cells.

### Determination of T cell subsets responsive to peptides of Erum5000A

**FIGURE 2:**
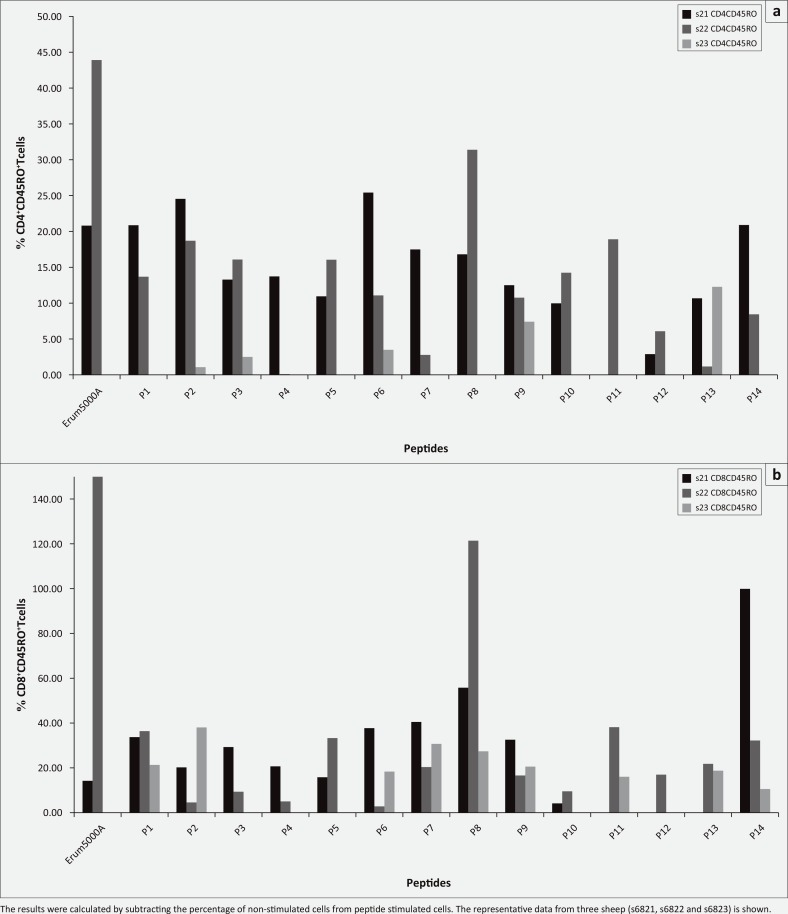
Percentage of cells expressing CD4+CD45RO+ (a) and CD8+CD45RO+ (b) surface markers after peripheral blood mononuclear cells stimulation with Erum5000A peptides for 48 h *in vitro*.

In an attempt to assess phenotype of PBMCs responsive to 14 Erum5000A peptides PBMCs from three sheep (s6821 s6822 and s6823) were used. CD4^+^ and CD8^+^ T cells expressing memory markers (CD45RO^+^) were measured by flow cytometry after 48 h stimulation. The percentages of CD4^+^ T cells expressing CD45RO^+^ was significantly induced by all peptides and varied between s6821 and s6822 with P2, P6, P8 and P14 showing best results (Figure 2). Whereas for PBMCs from s6823, only peptides P9 and P13 induced significant CD4^+^ T cells expressing CD45RO^+^. Similarly, the percentage of CD8^+^ T cells expressing memory markers (CD45RO^+^) was remarkably high and varied between sheep. Peptide P8 (aa 57–72) induced a high percentage of CD8^+^ T cells expressing memory markers (CD45RO^+^) in all three sheep followed by P14 (aa 105–116).

Intracellular IFN-γ staining analyses revealed that both CD4^+^ and CD8^+^ T cells from sheep 6821 produced high amounts of IFN-γ in response to P6, P8 and P11. Similarly, CD4^+^ and CD8^+^ T cells from sheep 6823 produced high amounts of IFN-γ in response to P8 (Table 6). In the case of other peptides lower IFN-γ production by both subsets was evident. These results also indicate that CD8^+^ T cells are the main producer of IFN-γ in the presence of P8.

**TABLE 6:** Percentage of CD4+ and CD8+ T cells producing IFN-γ after *in vitro* stimulation of peripheral blood mononuclear cells from immune sheep (6821 and 6823) at a concentration of 10 μg/mL. The % increase was compared to medium.

Antigen	s6821		s6823	
	
	CD4+IFN-γ+ % increase	CD8+IFN-γ+ % increase	CD4+IFN-γ+ % increase	CD8+IFN-γ+ % increase
5000A	3.9	3.5	1.7	0.9
P1	0.8	0.5	1.8	3.3
P2	1.7	1.6	0.0	0.7
P3	0.9	1.1	0.8	2.5
P4	1.5	1.5	0.0	0.3
P5	2.1	2.0	2.4	3.4
P6	5.4	5.0	1.0	2.9
P7	2.8	1.5	2.9	5.5
P8	3.6	3.9	8.5	10.2
P9	1.6	1.8	2.0	3.0
P10	2.3	3.1	0.7	1.2
P11	4.5	5.2	0.6	0.9
P12	2.7	2.6	1.7	2.6
P13	1.4	1.5	2.1	2.7
P14	1.6	2.4	2.3	3.5

Values ≥ 1 are positive.

### PCR-RFLP typing of the Ovar-DRB1 and BoLA-DRB3 genes from sheep

The polymorphism of the ovine MHC class II DRB1 second exon (*Ovar*-DRB1, Konnai *et al*. 2003) was studied in four sheep, (s147, s6821 s6822 and s6823) by PCR-RFLP and BoLA-DRB3 for three bovine (b8347, b8460 and b8404). When each amplified DNA product was cleaved by the restriction enzymes, different patterns were observed. Patterns, *Ovar*-DRB1 and BoLA-DRB3 alleles based on restriction endonuclease enzyme digestion are listed (Tables 7 and 8). Three different alleles (*0332 and *0323, *0333) were obtained for sheep s147. Sheep s6821 and s6823 has two different sets of alleles (*0109 and *0201) and (*0201 and *0801) respectively. Whereas s6822 has homologous alleles (*0201, *0201). The alleles identified for s6821, s6822 and s6823 were also reported by Konnai *et al*. (2003). For bovine, only one allele was similar to alleles reported by van Eijk *et al*. (1992) and the remaining alleles were not present in any of the published work. Therefore, new and unknown alleles were found in this study.

## Discussion

T cell responses characterised by the expression of IFN-γ are essential in protection against *E. ruminantium* infection (Totté *et al*. 1999). A DNA vaccine with four 1H12 ORFs induced IFN-γ and protection in sheep against a needle challenge with *E. ruminantium* Welgevonden but not against a tick challenge (Pretorius *et al*. 2008). Subsequently genome-based high through put technologies were then exploited to study *E. ruminantium* immunogenic antigens in an effort to improve this vaccine. Many *E. ruminantium* proteins of unknown function, and some of the membrane-associated proteins, secreted, transporters, particularly the ABC transport system and proteases were identified and immune characterised. Expressed recombinant proteins were assayed using NI immune sheep or bovine PBMC and they induced recall T cell responses characterised by elevation of IFN-γ *in vitro* and *in vivo* (Sebatjane *et al*. 2010), IFN-γ, TNF-α, GM-CSF, iNOS and TLR4 expression (Liebenberg *et al*. 2012).

These studies all used NI animals to screen for protective antigens. However, indications are that antigens that induce protection against natural and not artificial challenge should be identified for inclusion in a DNA vaccine. In this study we therefore investigated whether secreted *E. ruminantium* recombinant proteins would induce similar immune responses *in vitro* by PBMC from NI and TI animals. From a total of 27 secreted proteins (Liebenberg *et al*. 2012) three were selected for this study. Secreted proteins are regarded as promising vaccine candidates based on their ability to induce cell-mediated immunity characterised by production of IFN-γ (Carlisle *et al*. 2007). These proteins were successfully expressed in *E. coli*. They induced varied but significant proliferative responses and production of IFN-γ in PMBC from most animals tested. Recombinant proteins Erum5000 and Erum7760 induced the best response in PBMC from TI sheep whereas Erum8060 induced the best results in PBMC from NI sheep. The responses induced by all three proteins were also greater in cells from spleen and lymph nodes than PBMC from TI sheep. This is expected because these compartments have larger numbers of lymphocytes than PBMC improving the chance for antigen encounter with specific T cells (Blum & Pabst 2007).

The difference in immune responses obtained also suggests that the lymph node and spleen T cells may have distinct subsets of T lymphocytes (Langeveld, Gamadia & Ten Berge 2006). In addition, the spleen and LN cells of the tick infected animal produced the highest levels of IFN-γ protein, whereas the LN cells isolated from the needle infected animal remained unresponsive. This may be linked to the two different routes of infection that led to different homing patterns for the respective memory cells. This highlights the necessity to use TI animals to search for vaccine candidate antigens.

In addition to the cellular immune response, serum of NI immune animal detected the recombinant proteins (Figure 1-A1, supporting information) confirming that all proteins were secreted and exposed to the humoral immune system of the host during infection. However, when measuring IL-4 (associated with Th2 type responses) IL-4 production was not detected (results not shown) thus the antibodies detected could be opsonising antibodies (IgG2) associated with a Th1 response. Alternatively, the proteins may contain cross-reacting epitopes of a protein that induces a humoral immune response. It has been indicated that antibody-mediated immunity for intracellular parasites is not unusual (Doyle *et al*. 2006). Because the recombinant protein Erum5000 induced the best overall response it was equally divided and four recombinant fragment proteins Erum5000A, Erum5000B, Erum5000C and Erum5000D were expressed. Similar proliferative responses and production of IFN-γ were induced by the fragments in PBMCs isolated from the animals. However, the N-terminal fragment of Erum5000 rprotein Erum5000A induced the highest recall responses and was selected for epitope mapping.

Epitope mapping for this protein was done to identify vaccine candidates for incorporation into a multivalent vaccine. Epitope mapping of Erum5000 was determined using 14 overlapping peptides (P1–P14) spanning the fragment and PBMCs from NI cattle and TI sheep. The peptides that induced the best response in both cattle and sheep were P3, P5, P8 and P14. These peptides induced memory CD4^+^ and in particular CD8^+^ to proliferate and secrete IFN-γ.

**TABLE 7:** The polymerase chain reaction-restriction fragment length polymorphism patterns and *Ovar-DRB1* alleles obtained using nine restriction enzymes.

Sheep number	*Rsa*I	*Hae*III	*Sac*I	*Sac*II	*Dde*I	*Hin1*I	*Nci*I	*Eco*RI	*Bst*NI	*Ovar-DRB1* alleles
147	e	a	a	a	a	a	a	a	a	*0332 except for *Hae*II & *Nci*I
	f	a	a	a	a	a	a	a	a	*0323, *0333 except for *Hae*II
6821	f	f	b	a	a/c	b	a	a	a	*0109 except for DdeI
	g	d	a	a	a	b	a	a	a	*0201
6822	g	d	a	a	a	b	a	a	a	*0201
	g	d	a	a	a	b	a	a	a	*0201
6823	g	d	a	a	a	b	a	a	a	*0201
	a	a	a	a	b	a	a	a	a	*0801

a,b,c,e,f, restriction fragment length polymorphism patterns as identified by Konnai *et al*. (2003).

**TABLE 8:** The polymerase chain reaction-restriction fragment length polymorphism patterns and *BoLA-DRB3* alleles obtained using three restriction enzymes.

Bovine number	*Rsa*I	*Psu*I	*Hae*III	*BoLA-DRB3* alleles
8347	?	b	a	Unknown 1
	?	b	a	Unknown 1
8404	o	b	f	DRB3*1B
	?	b	e	Unknown 2
8460	c	b	?	Unknown 3
	f	a	?	Unknown 4

?, Does not correlate with known restriction patterns.

a,b,c,e,f, restriction fragment length polymorphism patterns as identified by van Eijk *et al*. (1992).

Each recombinant protein induced immune responses and it varied between animals. Similarly, peptides were recognised to various extents by immune animals. This can be expected because out bred animals were used for these assays. It has been indicated previously that MHC class II molecules are highly polymorphic and different alleles vary in their peptide binding specificity (Groothuis *et al*. 2005; Sommer 2006). The results obtained from MHC typing using ovine MHC II DRB1 and bovine BoLA II-DRB3 and PCR-RFLP highlighted the diversity between the animals because different alleles were found for each animal. This may also explain the variation of responses between sheep and bovine PBMCs. Nevertheless, the data confirm that heartwater immune sheep and cattle were exposed to the proteins and peptides during infection and that recall immune responses developed in the hosts.

Both CD4^+^ and CD8^+^ T cells are IFN-γ-producing lymphocytes that are required in the development of protective immunity against heartwater. The CD4^+^ T cells play a major role to both cell-mediated and humoral immunity and act through the production of IFN-γ, as helper cells for immunoglobulin secretion and as effector cells to activate macrophages. Cytotoxic CD8^+^ T cells limit bacteria replication by killing infected cells or by releasing cytokines that mediate intracellular killing of the pathogen through the production of iNOS (Yewdell & Haeryfar 2005). Previously, T cell growth factors (Mahan, Smith & Byrom 1994) and IFN-γ (Totté *et al*. 1996) have been shown to inhibit *E. ruminantium* growth *in vitro*. The effect of IFN-γ may be because of upregulation of MHC class I and II expression on monocytes leading to increased antigen presentation to immune cells, or by increased phagocytosis, reactive oxygen intermediates, nitric oxide and lysosomal enzyme production. Hence the role of both CD4^+^ and CD8^+^ T cells, and IFN-γ production in the development of protective immunity against heartwater should be a high priority.

In conclusion, we have demonstrated that predicted secreted *E. ruminantium* proteins induced a more robust Th1 type response by TI than NI animals, as indicated by proliferation of mononuclear cells (blood, spleen and lymph node) and higher production of IFN-γ. Furthermore, epitope mapping of Erum5000A identified four peptides that were recognised by immune PBMCs from both NI cattle and TI sheep. These epitopes require further investigation as components for inclusion in the multiepitope DNA vaccine against heartwater.

## Figures and Tables

**FIGURE 1-A1:**
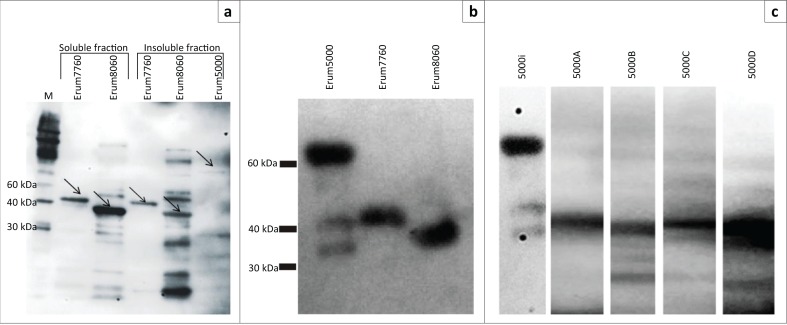
Western blot analysis of the whole protein rErum5000i and four fragments, Erum5000A, Erum5000B, Erum5000C and Erum5000D, Erum7760 and Erum8060. The purified recombinant proteins are expressed and purified using Champion^TM^ pET Directional TOPO^®^ expression system. Recombinant proteins were detected by using (a) immune sheep sera and (b and c) anti-His antibodies.

## Appendix 1

### Results

**TABLE 1-A1:** Primer sequences used for cloning of three open reading frames into pET vector.

Primer	Forward primer 5’-3’	Reverse primer 5’-3’
Erum7760	AAGGAACGTGTTGTAAAATCTGTTG	GTTATGGGACAATACATACCACTCAC
Eum5000	CTAAAGCTAATACAAATGATAACAAGATAAC	AAATTCATACTTCATACCCAACAATAGAAC
Erum8060	ATTAATACAAAAACAGATAACACAGTTAAAAC	TTCTGAATAATAATGGGATATATCATGTTTAAATTC
Erum5000A	GCTAAAGCTAATACAAATGATAACAAGATAAC	ATAAGAAGAATCAATAAATAACTGGGC
Erum5000B	CACCGGAAATATATCAATGGGTTATCAGGAAGG	CATCACGATCTGCATGTTCAAACAAGC
Erum5000C	CACCATTATAAAGTTGATAAATTAACAATAC	ATGTACCAACAGCAATACCTGATAAAC
Erum5000D	CACCCTGTTGCTTATGATAGTGTAATAGTTG	TCTAGAAAATTCATACTTCATACCCAACAATAGAAC

**TABLE 2-A1:** Fourteen 16 mer synthetic peptides spanning the N-terminal fragment of Erum5000 (348 bp). The peptides overlap each other by 8 aa.

Peptide number	1^st^ aa	aa sequence	Last aa
1	1	AKANTNDNKITVNTKK	16
2	9	KITVNTKKNHSDMKRV	24
3	17	NHSDMKRVVLRKLAKG	32
4	25	VLRKLAKGYHRIELGG	40
5	33	YHRIELGGQILSYAWC	48
6	41	QILSYAWCTDNVNQSQ	56
7	49	TDNVNQSQKHGVGVSG	64
8	57	KHGVGVSGILNVKSVS	72
9	65	ILNVKSVSENPNLGIS	80
10	73	ENPNLGISYGASLQIG	88
11	81	YGASLQIGVPAIKDKY	96
12	89	VPAIKDKYFIPTVKAY	104
13	97	FIPTVKAYNRGAQLFI	112
14	105	NRGAQLFIDSSY	116
